# “Is dopamine involved in Alzheimer's disease?”

**DOI:** 10.3389/fnagi.2014.00252

**Published:** 2014-09-25

**Authors:** Alessandro Martorana, Giacomo Koch

**Affiliations:** ^1^Clinica Neurologica-Memory Clinic, System Medicine Department, Università di Roma “Tor Vergata”Rome, Italy; ^2^Non Invasive Brain Stimulation Unit, Department of Behavioral and Clinical Neurology, Santa Lucia Foundation IRCCSRome, Italy

**Keywords:** Alzheimer's disease, dopamine, α7-nicotinic receptor, extrapyramidal signs, apathy

## Abstract

Alzheimer's Disease (AD) is a neurodegenerative disorder characterized by progressive cognitive decline and dementia. Recent advances indicate that AD pathogenesis appears more complex than its mere neuropathology. Changes in synaptic plasticity, neuronal disarray and cell death are pathways commonly recognized as pathogenic mechanisms of AD. It is thought that the altered metabolism of certain membrane proteins may lead to the production of amyloid (Aβ) oligomers that are characterized by an highly toxic effect on neurotransmission pathways, such as those mediated by Acetylcholine. The interaction of Aβ oligomers with these neurotansmitters systems would in turn induce cell dysfunction, neurotransmitters signaling imbalance and finally lead to the appearance of neurological signs. In this perspective, it is still debated how and if these mechanisms may also engage the dopaminergic system in AD. Recent experimental work revealed that the dopaminergic system may well be involved in the occurrence of cognitive decline, often being predictive of rapidly progressive forms of AD. However, a clear idea on the role of the dopamine system in AD is still missing. Here we review the more recent evidences supporting the notion that the dopaminergic dysfunction has a pathogenic role in cognitive decline symptoms of AD.

## Introduction

Alzheimer's disease (AD) is the most common form of human cognitive decline and dementia. AD is highly associated with aging and its pathological hallmarks, namely senile plaques (SP) and neurofibrillary tangles (NFT). It has been considered for a long time the result of an accelerated aging process of the brain since its pathological features resemble what we might find in aged brain (Becker et al., 2011; Ferrer, 2012). AD is currently defined as a progressive neurodegenerative disorder characterized by attention deficits, associated with progressive amnesia, apraxia, aphasia, agnosia, often associated with behavioral symptoms like anxiety, apathy, agitation, and in the later stages to hallucinations and psychosis (Lowe et al., 2009). Experimental and clinical evidences showed that AD is the consequence of the pathologic cleavage of a membrane protein, the amyloid precursor protein (APP), whose functions are still under study (Nalivaeva and Turner, 2013). Amyloid hypothesis postulates that APP undergoes impairment of physiological cleavage system due to unknown trigger(s), producing oligomeric toxic species, which are able to induce impairment of synaptic plasticity mechanisms, neuronal network disarrangement and cell death (Hardy and Selkoe, 2002). Oligomers are physiologically produced, and likely involved in cell signaling, and cleared either by proteolytic systems or in balance with monomers with protective function (Turner et al., 2003; Puzzo et al., 2008). An imbalance between production and clearance is supposed to lead to overproduction of oligomers, which altered stoichiometry could be responsible for cell degeneration and death (Jin et al., 2011; Puzzo and Arancio, 2013). Oligomers induce metabolic and morphologic changes in pyramidal neurons of the hippocampus and of the neo-cortex. Prefrontal and cingulate cortices together with hippocampus show changes since early stage of the disease and are responsible for cognitive decline symptoms (Sperling et al., 2010). Sub-cortical nuclei, serotonin- norepinephrine- and dopamine-containing, constitute the monoaminergic ascending innervating system that diffusely synapse onto hippocampal and neocortical neurons, with several modulatory effects on neuronal firing (Trillo et al., 2013). Recent neuro-pathological studies have established a link between morphological and functional changes occurring in the monoaminergic ascending system, particularly in norepinephrine and serotonin, and the pathophysiology of AD (Simic et al., 2009; Trillo et al., 2013). The progressive degeneration of these nuclei, that represents an early event, deprives hippocampal and cortical neurons from their critical influence. The progressive denervation could be the cause of cognitive decline symptoms presenting with AD. In association to cognitive decline symptoms about 35–40% of AD patients present with extrapyramidal signs, supporting the idea that dopamine (DA) containing neurons undergo degenerative changes (Lopez et al., 1997). Neurons forming the nigrostriatal pathway showed several pathologic changes like NFT, Aβ plaques, neuropil threads, neuronal loss and also decrease in DA content, all changes suggesting the clear involvement of DA in the pathophysiology of cognitive decline and non-cognitive symptoms of AD (Rudelli et al., 1984; Gibb et al., 1989; Braak and Braak, 1990; Selden et al., 1994; Storga et al., 1996; Burns et al., 2005). Despite that, the direct involvement of DA in AD still remains debated (Lopez et al., 1997). Aim of this work is to define the possible role of DA in the course of AD.

## Dopamine system anatomy

Dopamine (DA) containing neurons are mainly located in the midbrain. They can be detected in A8, A9, A10 areas corresponding to the retrorubral field (A8), the Substantia Nigra pars compacta (SNc) (A9) and the Ventral Tegmental Area (VTA) (A10). Each area projects to different brain regions, exerting different functions. SNc gives rise to the meso-striatal pathway, targeting the medium spiny projection neurons of the caudate and putamen nuclei (Lammel et al., 2011; Bolam and Pissadaki, 2012). This pathway together with the corticostriatal glutamatergic projections (from the sensory-motor cortex) are involved in the control of voluntary movements. VTA instead gives rise to the meso-cortico-limbic pathway, targeting hippocampi, cerebral cortex and the nucleus accumbens. DA terminals together with glutamatergic projections from amygdala, hippocampus and prefrontal cortex (PFC), are involved in the control of volition and reward (Haber and Fudge, 1997; Haber and Knutson, 2010). The A8 field represents the 4–5% of DA neurons, whose targets haven not been fully discovered yet. In general, pathological alterations of the meso-striatal pathway are generally associated to the development of extrapyramidal motor deficits, while the involvement of the meso-cortico-limbic pathway is responsible for cognitive, behavioral signs. Both could be involved in progression of AD.

## Dopamine system in Alzheimer's disease

Brain cell types show different sensitivity to toxic Aβ oligomers, being cholinergic the most sensitive, followed by serotonin-ergic. GABA-ergic cell types are less vulnerable to Aβ pathology in comparison with the latter. DA-ergic neurons show intermediate features, having anyhow an unclear relationship with Aβ pathology (Kar et al., 2004). The involvement of DA in AD has been investigated for a long time and is still under debate (Attems et al., 2007; Portet et al., 2009; Trillo et al., 2013). DA system undergoes several changes during physiological aging process. In general, decreased release of DA from its terminals, reduced DA receptor expression in particular D2 subtypes, reduced DAT expression in caudate putamen, hippocampus and frontal cortex of humans are features commonly observed in the brain during brain aging (Volkow et al., 1994; Bäckman et al., 2010). The association with a physiologically reduced glutamate release from frontal and prefrontal cortices, hippocampi and amygdala would induce further decrease of DA release, inducing hypo-activity, gait disturbances and decline of executive functions. The occurrence of apathy, a negative prognostic sign in both elderly and AD, is suggested to be the consequence of the impairment of DA transmission observed during normal aging as well (Robert et al., 2010). Decline of executive functions and apathy are predictive of “unsuccessful aging” and are considered indicators of frailty among the elderly (Yates, 2002; Depp and Jeste, 2006). Similarly, in AD patients, apathy and executive dysfunction have negative prognostic value and are considered predictive of faster cognitive decline and life shortening (Musicco et al., 2010; Koch et al., 2013). Extrapyramidal signs like bradykinesia, face masking, tremors, gait disturbances, may occur early or more commonly in the later stages of AD, particularly if the patient is under treatment with neuroleptics (Portet et al., 2009; Vilalta-Franch et al., 2013). In this view in both physiological and pathological processes the appearance of DA dysfunction represents a negative prognostic evolution. Thus, the earlier the impairment of DA system occurs, the fastest the cognitive decline goes. These considerations are in contrast with early neuropathological studies that found no neuronal loss but only degenerative changes like Lewy bodies or (NFT). These findings suggest that extrapyramidal signs (EPS) appearance could be dependent on extra-nigral mechanisms, given that there is a lack of severe pathology of DA system in AD (Murray et al., 1995; Burns et al., 2005; Attems et al., 2007). Although these data are consistent with *in vivo* imaging and neuropathologic studies on meso-striatal pathways in AD and Lewy bodies dementia (DLB) patients (Colloby et al., 2012), the involvement of nigrostriatal pathway with Lewy bodies and alpha sinucleinopathy, generally associated to the occurrence of DLB, has been observed in about half of AD patients as well as 30% of aged healthy controls (Jellinger, 2004). Therefore, the presence of such pathological changes have pathogenetic and clinical relevance to be still elucidated in cases of DLB as well as of AD (Jellinger, 2009; Huang and Halliday, 2013). However, several recent experimental evidences renewed interest on DA involvement in AD pathogenesis. Indeed, experimental data from transgenic mice AD showed how the DA-ergic pathology and amyloid deposition are closely related, suggesting a causative role for amyloid on dopamine dysfunction (Perez et al., 2005). Moreover, the restoration of DA transmission was demonstrated to play a role in memory and learning in a mouse model of AD, strengthening the central role of DA in cognitive tasks (Ambrée et al., 2009; Guzmán-Ramos et al., 2012). This has been recently associated to a demonstrated protective role, having DA an anti-amyloidogenic and anti-oxidant effects in mice brain (Himeno et al., 2011). Moreover, although the pathological substrates of extrapyramidal signs remains debated, recent study on AD with parkinsonian features showed that motor impairment is the consequence of nigrostriatal pathology, leading authors to conclude that prominent subcortical involvement occur in these patients, a process apparently not related to the Braak stages (Horvath et al., 2014). Accordingly, signs of tau pathology in brain stem nuclei, becoming increasingly involved with AD progression has been proposed (Grinberg et al., 2009; Simic et al., 2009; Attems et al., 2012). More details on DA transmission system in both cortical and sub-cortical brain areas, which have never been observed before, have been recently discovered. In particular, a markedly reduced expression of both subtypes of DA receptors, D1-like and D2-like has been observed in prefrontal cortex and in hippocampus of AD patients (Kemppainen et al., 2003; Kumar and Patel, 2007). Interestingly, although the dorsal striatum is relatively spared in AD, its ventral homologous, the nucleus accumbens, is highly affected. A decreased dopamine receptor expression, particularly D2-like, a reduced expression of Dopamine Transporter and Tyrosine Hydroxylase Enzyme were observed in this nucleus (Rinne et al., 1986; Allard et al., 1990; Murray et al., 1995; Joyce et al., 1997). Furthermore, recent imaging studies showed atrophy of this nucleus in a cohort of late onset AD patients, but not in the early-onset AD patients (Pievani et al., 2013). Electrophysiological studies performed on AD patients showed unexpected positive effects of DA drugs on cortical neurotransmission and synaptic plasticity mechanisms and also on cognitive performances, suggesting a possible therapeutic effects for these drugs in the treatment of AD (Martorana et al., 2009, 2013; Koch et al., 2014). All these data strengthen the hypothesis of DA involvement in AD, without answering the fundamental question: how DA dysfunction occurs?

## Dopamine dysfunction hypothesis

Amyloid hypothesis remains the leading hypothesis to interpret the neurotransmitters dysfunctions occurring in AD since early phases of the disease. In this section we suggest at least two possible pathogenetic mechanisms that might be responsible for the DA-ergic dysfunction since early phases of the disease. Aβ oligomers show highly toxic and deleterious effects on neuronal arrangement and neurotransmission, and cause neuronal death of the neocortex and hippocampi (Palop and Mucke, 2010). In experimental models Aβ oligomers have been postulated to induce pathological synaptic plasticity, with altered glutamate transmission, impairment of the long term potentiation, prolonged and pathological long term depression, with subsequent spine shrinkage, and activation of enzymatic pathways leading to apoptosis and cell death (Palop and Mucke, 2010). All these changes occur at the level of glutamatergic synapses, obeying to the hypothesized excitotoxic hypothesis of AD. Aβ oligomers are also able to induce multiple effects on acetylcholine neurons transmission, impairing physiological cell metabolism, transmitter synthesis and release, until cell degeneration (Kar et al., 2004; Ferrer, 2012; Esposito et al., 2013). Beside cholinergic neurons degeneration, several Aβ-mediated effects are secondary to its binding to cholinergic receptors, particularly with nicotinic receptors (Schliebs and Arendt, 2011). Neuronal nicotinic acetylcholine receptors (nAChR) are a family of ligand-gated pentameric cation channels constituted by a combination of α and β subunits (α2-6 and β 2-4). The other major neuronal nAChRs subtype contain the α7 subunit (bungarotoxin sensitive). nAChRs can be located on the presynaptic terminal where they regulate post-synaptic transmission and modulate pre-synaptic transmitter release. Post-synaptic localization mediates depolarization of neurons (Ni et al., 2013). The main role of nAChRs in the brain is to regulate transmitters release and allow Ach to participate in attention, learning and memory functions through the α4β 2 and α7 receptors. The α7AChRs are located predominantly, but not exclusively, at pre-synaptic sites of (PFC), hippocampus, thalamus, serotonin-raphe nucleus and DA neurons of the VTA. Pre-synaptic localization of these receptors increase the probability of neurotransmitters release, while post-synaptic localization through the intracellular Ca^2+^ increase activates intracellular metabolic pathways, which are related to neuronal homeostasis, synaptic plasticity, learning, and memory (Alkondon and Albuquerque, 2002). Several lines of evidence indicate that Aβ oligomers have the ability to bind to α7 nAChRs (Schliebs and Arendt, 2011; Posadas et al., 2013). The high affinity interaction with these receptors can cause intracellular Ca^++^ increase and activation of the ERK-MAPK cascade with positive modulatory effects on synaptic plasticity mechanisms and functions like memory and learning in hippocampal neurons. Conversely, the prolonged exposure to high Aβ oligomers can reverse such a positive effect inducing the formation of LTD and impairment of synaptic mechanism machinery and of learning and memory. Such conflicting effects are likely dependent on concentration and stoichiometry of the Aβ oligomers. Very low concentrations (picomolar) are able to enhance memory and synaptic functions, on the contrary higher concentrations (nanomolar) and prolonged exposure to them can disrupt mechanisms of synaptic plasticity and memory functions (Puzzo and Arancio, 2013). Since that, it is conceivable to suppose that DA dysfunction in AD, at least in early stages, could be the consequence of Aβ peptides production and prolonged interaction with α7 nAChRs, overwhelming the nicotinic receptor function, and interfering with the physiological DA functioning. Thus, prolonged exposure to Aβ would progressively impair the physiological release of glutamate and of GABA reducing the possibility of DA release in prefrontal cortex and hippocampus, contributing to the impairment of attention, memory, and executive functions. The progressive decrease of glutamate release from the PFC would in turn reduce the stimulus for DA release also in the NAcc, being responsible for appearance of apathy and motor hypoactivity (Wang et al., 2000; Posadas et al., 2013). In the later stages, following prolonged dysfunctioning of nicotinic receptors in the SN, the inactivation of ERK-MAPK pathways would favor cytoskeletal tau protein hyperphosphorylation process (Wang et al., 2000) with neuronal degeneration and cell death. The persistence of this mechanism could be responsible for SNc neurons degeneration and for the appearance of extrapyramidal signs. Alternative hypothesis come directly from recent experimental evidences on misfolding and pathological aggregation of neuronal proteins. Recently has been shown that Aβ oligomers could be able to induce under pathological conditions the aggregation of other proteins like alpha-synuclein (α-syn). Experimental evidences showed both *in vivo* and *in vitro* that Aβ was able to interfere with α-syn inducing conformational changes and neurodegeneration (Iqbal et al., 2000; Mitchell et al., 2011). In physiological conditions soluble Aβ can be identified in the cytosolic fraction, in endosomes and in endoplasmic reticulum (Starkstein et al., 2009). Similarly, monomeric α-syn is primarily found in the cytosolic fraction and is associated with synaptic vescicles where it is supposed to play a role in neurotransmitters release (Richard et al., 2012). Under pathological conditions, both aggregated Aβ and α-syn might associate with membranes and accumulate in caveolae. Hence, it is suggested that during early phases of AD, Aβ interact with α-syn bound to membrane and inducing the aggregation of this protein and the formation of pentamers and hexamers with ring-like structure that associate to membrane forming pores. These structures would form ion-permeable channels, particularly for Ca^2+^ that may play a role in the mechanisms of neurodegeneration (Van der Vlies et al., 2009; Iqbal et al., 2013). Such interesting hypothesis that proposed for DLB cases has been suggested also for AD and PD cases though needs to be further investigated however. Therefore, the production of pathologic Aβ would be responsible for DA-ergic pathology and as a consequence for extrapyramidal behavioral/motor deficits in early phases of AD. Such mechanism could be reasonably responsible also for rapid progression of cognitive decline.

## Clinical features of dopamine-dependent symptoms

Apathy is the most common neurobehavioral symptom associated to AD. EPS are associated to AD disease progression, often related to neuroleptic assumption, and are definitely a negative prognostic sign. Both apathy and EPS are secondary to DA dysfunction and their appearance is a sign of disease progression for individuals with mild cognitive impairment, as well as for AD patients (Iqbal et al., 2000; Mitchell et al., 2011). Both clinical conditions are associated to Aβ burden, and both could co-exist in the same individual. Although frequently described, not all individuals with AD diagnosis may experience apathy and EPS symptoms during their life. About half of AD patients presents with symptoms of apathy, and one third with EPS in early-mid stage of the disease. In the later stages of disease, accomplice physiological aging, EPS are more clearly and easily diagnosed in almost all individuals with AD. Nowadays, Aβ levels are easily detectable in CSF of AD individuals and currently used for diagnostic purposes. Due to its low threshold, Aβ levels that are so useful to individuate cognitively compromised individuals, unfortunately cannot be used to evaluate whether the oligomer-mediated toxicity has already reached cortical and/or subcortical nuclei. Results from recent neuropathological and clinical studies pointed out the need to individuate different subgroups of AD patients, possibly characterized by a clinical hallmarks to obtain patients stratification for clinical purposes (Starkstein et al., 2009; Richard et al., 2012). These would help to better individuate patients with more prominent cholinergic/glutamatergic deficits with respect to those presenting with symptoms of DA dysfunction and so on. Recently published clinical studies described features of AD patients through their different degrees of progression. Clusters of AD diagnosed patients were identified and characterized by different degree of executive functions impairment, high levels of CSF tau and phosphorylated tau with low levels of Aβ 1-42, presence of apathy, limited or absent response to pharmacological treatment with cholinesterase inhibitors (Van der Vlies et al., 2009; Mitchell et al., 2011; Iqbal et al., 2013; Koch et al., 2013; Horvath et al., 2014). Among them the presence of both apathy and very high levels of CSF tau protein were related to more rapid progression of cognitive decline (Wallin et al., 2010; Schmidt et al., 2011). Tau pathology is considered the hallmark of neurodegeneration, and it is not specific for AD. Interestingly, recent literature showed the presence of tau related pathology in brainstem of AD. These authors claimed that a certain degree of tau pathology in the monoaminergic tract could represent the substrate for more intense neurodegeneration in AD cases (Attems et al., 2007, 2012; Simic et al., 2009; Trillo et al., 2013). This would indicate that a subset of AD individuals could be more prone to develop DA-deficit related symptoms. Moreover, the monoaminergic tract have been shown to be able to control the physiologic amyloid-synthesis process (Himeno et al., 2011). It is conceivable to suppose that the progressive dysfunction and/or degeneration of monoaminergic neurons could represent a step forward for increased amyloid production and for amyloid-related degeneration of neurons.

## Conclusions

Different degree of DA dysfunction can occur during any phases of AD. The most important symptoms related to DA dysfunction are the presence of apathy and EPS. The occurrence of these symptoms could be related to pre-existing brainstem pathology. Their burden can vary in relation to the age of an individual that remains the most important risk factor associated to AD. In general, DA related symptoms appearance is related to increased intensity of neuronal degeneration and to faster cognitive decline. In our recent experience the use of dopaminergic drugs, in particular of the DA- D2-agonists rotigotine, showed beneficial effects on some cognitive domains in AD patients (Koch et al., 2014). The use of these drugs was well tolerated with no relevant behavioral side effects. Future clinical trials are needed to verify the potential therapeutic effectiveness of dopaminergic drugs in AD patients.

### Conflict of interest statement

The authors declare that the research was conducted in the absence of any commercial or financial relationships that could be construed as a potential conflict of interest.
